# Calcium imaging and analysis of the jugular-nodose ganglia enables identification of distinct vagal sensory neuron subsets

**DOI:** 10.1088/1741-2552/acbe1e

**Published:** 2023-03-15

**Authors:** Tomás S Huerta, Bilal Haider, Richard Adamovich-Zeitlin, Adrian C Chen, Saher Chaudhry, Theodoros P Zanos, Sangeeta S Chavan, Kevin J Tracey, Eric H Chang

**Affiliations:** 1 Laboratory for Biomedical Sciences, Institute for Bioelectronic Medicine, Feinstein Institutes for Medical Research, Northwell Health, Manhasset, NY, United States of America; 2 Donald and Barbara Zucker School of Medicine at Hofstra/Northwell, Hempstead, NY, United States of America; 3 Institute of Health System Science, Feinstein Institutes for Medical Research, Northwell Health, Manhasset, NY, United States of America; 4 Elmezzi Graduate School of Molecular Medicine, Manhasset, NY, United States of America

**Keywords:** vagus nerve, peripheral nervous system, single-photon fluorescence imaging, miniscope, GCaMP

## Abstract

**Objective.:**

Sensory nerves of the peripheral nervous system (PNS) transmit afferent signals from the body to the brain. These peripheral nerves are composed of distinct subsets of fibers and associated cell bodies, which reside in peripheral ganglia distributed throughout the viscera and along the spinal cord. The vagus nerve (cranial nerve X) is a complex polymodal nerve that transmits a wide array of sensory information, including signals related to mechanical, chemical, and noxious stimuli. To understand how stimuli applied to the vagus nerve are encoded by vagal sensory neurons in the jugular-nodose ganglia, we developed a framework for micro-endoscopic calcium imaging and analysis.

**Approach.:**

We developed novel methods for *in vivo* imaging of the intact jugular-nodose ganglion using a miniature microscope (Miniscope) in transgenic mice with the genetically-encoded calcium indicator GCaMP6f. We adapted the Python-based analysis package Calcium Imaging Analysis (CaImAn) to process the resulting one-photon fluorescence data into calcium transients for subsequent analysis. Random forest classification was then used to identify specific types of neuronal responders.

**Results.:**

We demonstrate that recordings from the jugular-nodose ganglia can be accomplished through careful surgical dissection and ganglia stabilization. Using a customized acquisition and analysis pipeline, we show that subsets of vagal sensory neurons respond to different chemical stimuli applied to the vagus nerve. Successful classification of the responses with a random forest model indicates that certain calcium transient features, such as amplitude and duration, are important for encoding these stimuli by sensory neurons.

**Significance.:**

This experimental approach presents a new framework for investigating how individual vagal sensory neurons encode various stimuli on the vagus nerve. Our surgical and analytical approach can be applied to other PNS ganglia in rodents and other small animal species to elucidate previously unexplored roles for peripheral neurons in a diverse set of physiological functions.

## 1. Introduction

Sensory signals in the body are relayed to the brain via peripheral nerves, such as the vagus nerve. These sensory afferent signals travel from innervated organs or tissues and are transmitted within the peripheral nervous system (PNS), a complex network of nerves that reside outside of the brain and the spinal cord. To understand how sensory signals are encoded in the nervous system, electrophysiological techniques are typically used to record from neuronal cell bodies. Within the PNS, the primary cell bodies reside within peripheral ganglia that are distributed throughout the body, but are often located in difficult to access regions where soft tissue predominates or in close proximity to major blood vessels. This makes it challenging to gain surgical access and record from some of these PNS ganglia *in vivo* using traditional neurophysiological approaches, such as rigid electrodes or probes.

Standard electrophysiological approaches have relied on electrical recordings via intracellular patch-clamping or the use of extracellular microelectrodes; techniques that are typically limited to recording the activity of single neurons or small groups of neurons (Buzsaki 2004). Recent advances in neuroscience, such as Neuropixels probes, have increased channel counts and expanded possibilities for large-scale *in vivo* electrophysiology but rely on penetrating probes designed for brain tissue (Jun *et al* 2017, Steinmetz *et al* 2018, 2021). An emerging and potentially complementary approach is ‘optical electrophysiology’ involving the imaging of calcium and voltage reporters that read out neuronal activity with high sensitivity (Akerboom *et al* 2012, Fan *et al* 2020). By using calcium imaging as a proxy for neuronal activity, techniques such as micro-endoscopic imaging enable the recording of neuronal activity over large populations of cells with cellular resolution (Aharoni and Hoogland 2019). The expression of these activity reporters can be selective for genetically identified populations of neurons through either transgenesis or Cre-mediated viral recombination (Zariwala *et al* 2012). The emergence of optical electrophysiology provides unique opportunities to access neuronal populations that prove difficult for more traditional electrode-based neurophysiological techniques (Emiliani *et al* 2015, Oh *et al* 2019). However, this approach also presents unique challenges related to fluorescence imaging and the increased computation required to interpret the resulting data (Robbins *et al* 2021).

Optical electrophysiology has thus far primarily been applied to monitor neurons in the brain, where skull-mounted miniature microscopes and endoscopic lenses provide a stable environment for imaging (Smedemark-Margulies and Trapani 2013). A relatively unexplored application of this technology has been for examining neuronal activity in the PNS, which is responsible for a complex range of physiological functions including somatosensation, organ homeostasis, interoceptive signaling, and immunity (Chavan *et al* 2017, Umans and Liberles 2018, Jakob *et al* 2021). The vagus nerve and other peripheral nerves connect the brain to the body with a complex neuroanatomical circuit that is just starting to be revealed in fine molecular and genetic detail (Kupari *et al* 2019, Zhao *et al* 2022). Vagus nerve fibers transmit a diverse array of sensory signals that are vital to proper physiological function. In addition to mediating major autonomic reflexes and vital functions, such as respiration and heart rate, the vagus nerve also transmits sensory afferent information related to chemical, temperature, mechanical, and noxious stimuli (Prescott and Liberles 2022). To better understand how sensory neurons encode various chemical stimuli applied to the vagus nerve, we use optical electrophysiology to record from vagal sensory neurons *in vivo*.

In this study, we describe a surgical procedure and approach to identify, isolate, and stabilize the jugular-nodose ganglia in mice for *in vivo* calcium imaging using a single-photon epifluorescence Miniscope (Cai 2016, Aharoni and Hoogland 2019). Accessing and stabilizing the jugular-nodose ganglia has been a challenge due to its location in the body and its close proximity to deeply embedded tissues and major blood vessels (Han and de Araujo 2021). Prior efforts to monitor neural activity in peripheral ganglia have focused on dorsal root ganglia (DRG) or have utilized *in vitro* approaches (Crosson and Talbot 2022). Because there are major differences in the signal-to-noise of the jugular-nodose ganglia fluorescence signal, compared to standard intracranial recordings, we then outline how to implement and modify Calcium Imaging Analysis (CaImAn), a Python-based analysis pipeline (Giovannucci *et al* 2019), to accurately extract calcium transients from raw fluorescence intensity data. Using this customized analysis pipeline, we demonstrate how features of the resulting calcium traces can be calculated and linked to activation by specific chemical stimuli applied to the vagus nerve.

## 2. Materials and methods

### 2.1. Overview

Experiments were performed on Vglut2-GCaMP6f mice (aged 2–8 months, ~20–30 g; bred in house) anesthetized with isoflurane (1.75% isoflurane at an oxygen flow of 1 l min ^1^). Prior to imaging experiments, animals were group-housed on a 12:12 h reverse light/dark cycle on an *ad libitum* chow diet. The jugular-nodose ganglia complex was surgically isolated from the submandibular area of the mouse, leaving the connections to the periphery intact. The jugular-nodose ganglion was placed on a stable mesh retractor bathed with saline. Calcium imaging experiments were performed by lowering a Miniscope (V3.2) onto the isolated jugular-nodose ganglion and visualizing a plane of active neurons. Several agonists were sequentially applied directly on the exposed vagus nerve, with alternating applications of saline wash. Experiments were concluded with the application of KCl as a positive control. Region of interest (ROI) detection and calcium transient plotting were performed with a custom CaImAn pipeline followed by a custom Python post-processing pipeline. Finally, vagus nerve and jugular-nodose ganglia complex samples were harvested and fixed in paraformaldehyde for subsequent immunohistochemistry.

### 2.2. Animals

Experiments used in this study were carried out using adult mice (male and female) between 2 and 8 months of age. All experiments were performed under protocols approved by the Institutional Animal Care and Use Committee of the Feinstein Institutes for Medical Research and in strict adherence to the NIH Guide for the Care and Use of Laboratory Animals.

To monitor the neural activity of individual sensory neurons *in vivo*, we created Vglut2–GCaMP6f mice by crossing homozygous Vglut2-ires-cre (Jax# 028863) mice with homozygous Ai95D, also known as ROSA-GCaMP6f (Jax# 028865) mice. In Vglut2–GCaMP6f offspring, Cre recombinase expression is directed to excitatory glutamatergic neurons, where the floxed-STOP cassette for a GCaMP6 fast variant calcium indicator (GCaMP6f), inherited from the Ai95D parent, is trimmed. This leads to selective EGFP fluorescence in glutamatergic soma observed following calcium binding, such as during neural activation.

### 2.3. Nodose isolation and stabilization

**Figure 1.**
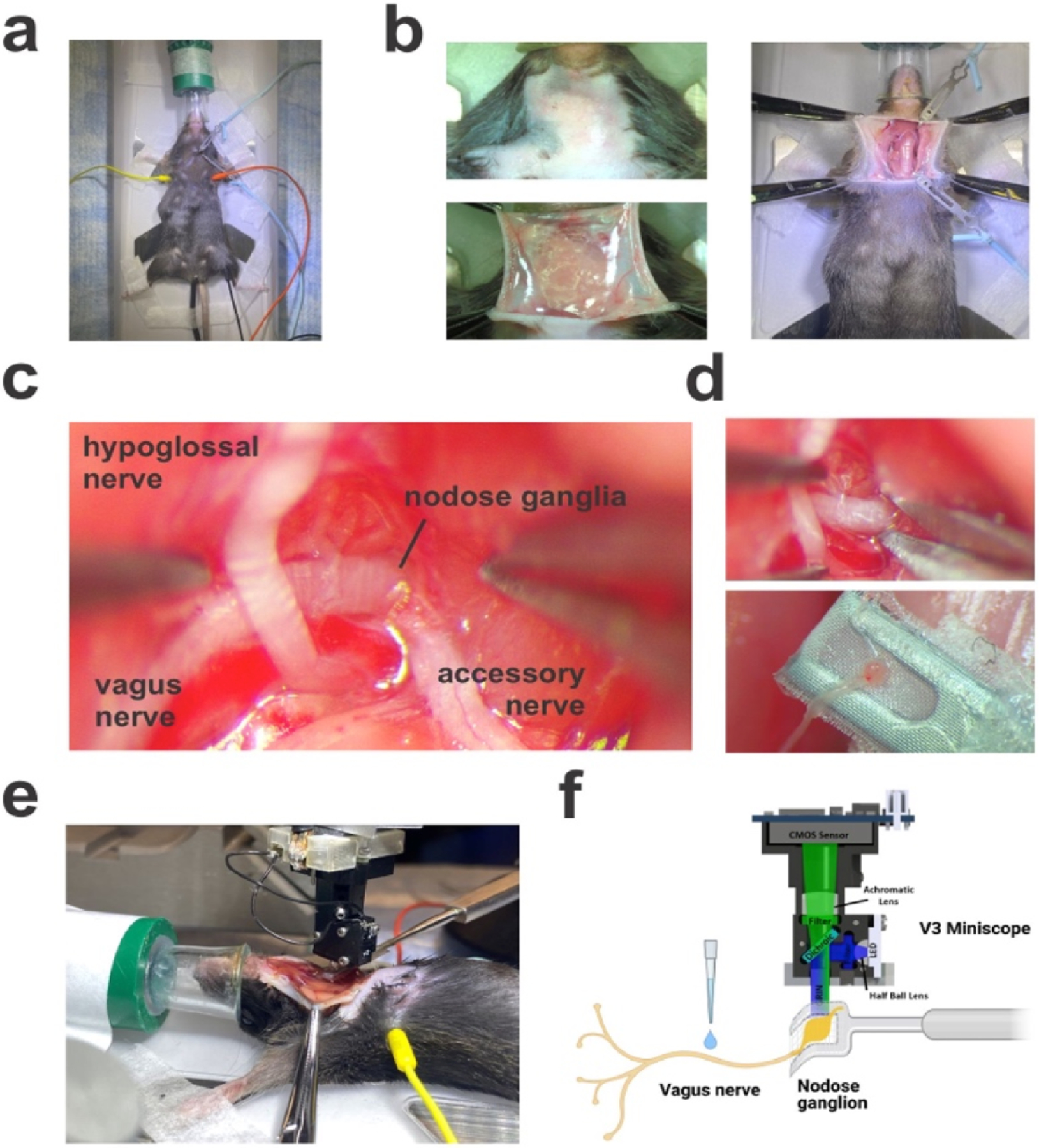
Surgical preparation for jugular-nodose ganglia imaging. (a) Pre-surgical preparation of the mouse. The mouse was anesthetized with 1.75% isoflurane at 1 l min ^1^ and connected to a small animal physiological monitoring system. (b) The submandibular area of the mouse was dehaired with Nair and cleaned with ethanol swabs. A midline incision was made, and four hemostats were pulled taut creating a surgical window. Once the salivary glands were separated, two elastic retractors are set to pull on the left masseter muscle and left sternohyoid muscle. (c) The jugular-nodose ganglia were visualized beneath the intersection of the hypoglossal nerve and the vagus nerve. (d) A pair of spring micro dissecting scissors was used to sever the brainstem adjoining projections of the jugular-nodose ganglia, leaving the projections to the periphery intact. The jugular-nodose ganglia were then placed on a stable retractor with a mesh covering soaked in saline. This preparation leaves the left vagus nerve exposed and open for application by dropping liquid stimuli. (e) The Miniscope was then lowered onto the jugular-nodose ganglia and stimuli were applied directly to the vagus nerve. (f) Schematic overview of the calcium imaging preparation.

All surgical procedures were conducted using aseptic techniques. Animals were administered isoflurane anesthesia through a nose cone in the supine position with an oxygen flow of 1 l min ^1^ at isoflurane 1.75% (figure 1(a)). The mouse was positioned on a small animal physiological monitoring system with integrated heating to maintain body temperature (Harvard Apparatus, Holliston, MA). An appropriate depth of anesthesia was assessed by the toe pinch reflex. The hair was chemically removed from the cervical region with Nair (Church & Dwight, Ewing Township, NJ), and then sterilized with 70% ethanol swabs (figure 1(b), Fisher Scientific, Waltham, MA).

A pair of micro dissecting scissors (RS-5912SC Roboz, Gaithersburg, MD) was used to make a midline incision in the submandibular area from the sternum to beneath the chin. Four hemostatic forceps RS-7111, Roboz, Gaithersburg, MD) were used to pull the corners of the surgical window. Two blunt retractors (18200-07 and 18200-09, Fine Science Tools, Foster City, CA) were used to pull the masseter muscles cranially and the sternohyoid muscle laterally. (figure 1(b)). A No.7 forceps (RS-5047, Roboz, Gaithersburg MD) in the main hand and a No.7 Vessel Dilation forceps (RS-4927, Roboz) in the off-hand were used to tease apart the connective tissue above the intersection of the left vagus nerve and the hypoglossal nerve. The jugular-nodose ganglion was then visualized under a branch of the carotid artery (figure 1(c)). A pair of micro dissecting spring scissors (RS-5602, Roboz) was used to excise the nodose on the rostral side. The vagus nerve was followed down near the base of the neck, and isolated from the carotid bundle. Pharyngeal and laryngeal vagal branches were severed, and the vagus nerve was carefully pulled through the trachea muscle and lifted out of the surgical cavity to be placed onto a custom mesh-covered retractor cut from 40 m EZFlow cell strainer (figure 1(d); Foxx Life Sciences, Salem NH). Note that it is important to minimize the amount of movement and physical force on the nerve as there are mechanoreceptors and other pressure-sensitive receptors that may be activated during this step. Following placement on the retractor, the jugular-nodose ganglion was bathed in saline, and a Miniscope (Cai 2016) was lowered onto the ganglion using a Stoelting stereotaxic frame arm (figures 1(e) and (f)). A full list of surgical tools, materials, and reagents is provided in table 1.

### 2.4. Utilizing the CaImAn pipeline

To process our raw fluorescence data, we used the open-source analysis pipeline Calcium Imaging Analysis (CaImAn), which is a semi-automated software suite implemented primarily in Python (Giovannucci *et al* 2019). Prior to CaImAn analysis, compressed Miniscope video files were converted into separate 1000 frame tagged imaged file format (TIFF) files. If necessary, the field of view was cropped to minimize the severity of the edge effect with the border of the ganglion complex and the dark background. We compressed the size of our field of view (752 280 px) in half (376 240 px) so that the neurons were approximately ~12 pixels large in diameter. Outputs from CaImAn are calcium traces, on which further quantification is performed through a custom analysis pipeline.

### 2.5. Immunohistochemistry

Following each imaging experiment, the jugular-nodose ganglia were excised and fixed in 4% paraformaldehyde (PFA) solution at 4 °C overnight and then transferred to 30% sucrose solution. Following embedding in optimal cutting temperature compound, tissue sections (10 m thickness) were prepared on a Leica CM1850 cryostat. Tissues were then incubated in blocking solution (10% normal donkey serum; NDS) and 0.3% Triton X-100 in 1X PBS. for 1 h at room temperature. Next, tissues were incubated with primary antibodies: vanilloid R1/TRPV1 antibody (Novus, NB100-98886, 1:250), anti-TRPA1 antibody (Millipore Sigma, ABN1009, 1:500), anti-PGP9.5 antibody (Genetex, GTX10410, 1:250), and anti-neurofilament heavy polypeptide antibody (Abcam, ab7795, 1:250) diluted in 0.3% Triton-100 and 10% NDS with 1 PBS overnight at 4 °C. Next, tissues were rinsed with 10x PBS-Tween 20 (0.1 M PBS, 0.5% Tween 20, pH 7.4) and incubated with secondary antibodies: Alexa Fluor 680 donkey anti-mouse (Invitrogen, A10038, 1:250), CF 647 goat anti-guinea pig (Biotium, 20495, 1:250), and CF 594 donkey anti-rabbit IgG polyclonal antibody (Biotium, 20152-1, 1:250)) diluted in 0.3% Triton-100 and 10% NDS with 1 PBS overnight at 4 °C. Lastly, tissues were rinsed with 10 PBS-Tween 20 and coverslipped with DAPI Fluoromount mounting medium (Southern Biotech, Birmingham, AL, US). Fluorescent images were acquired using a Zeiss LSM 900 confocal microscope.

### 2.6. Quantification and statistics

Data are presented as the mean ± standard error of the mean, unless otherwise indicated. Statistical comparisons were performed using Prism 9.0 software (GraphPad, San Diego, CA). Group differences were assessed using the non-parametric Kruskal–Wallis test, with *P* < 0.05 considered statistically significant.

## 3. Results

### 3.1. Miniscope fluorescence imaging

Following surgical isolation and stabilization of the jugular-nodose ganglion, the Miniscope-DAQ recording software was set to the following parameters for focus adjustment: 70 LED exposure, 3.5 gain, 20 FPS, 0.80 alpha, 0.10–0.15 beta. The ganglion was bathed in 200 l of saline solution This step maintains cellular viability and creates a continuous liquid medium for the Miniscope light excitation to travel and GCaMP6f emission to be detected.

**Figure 2.**
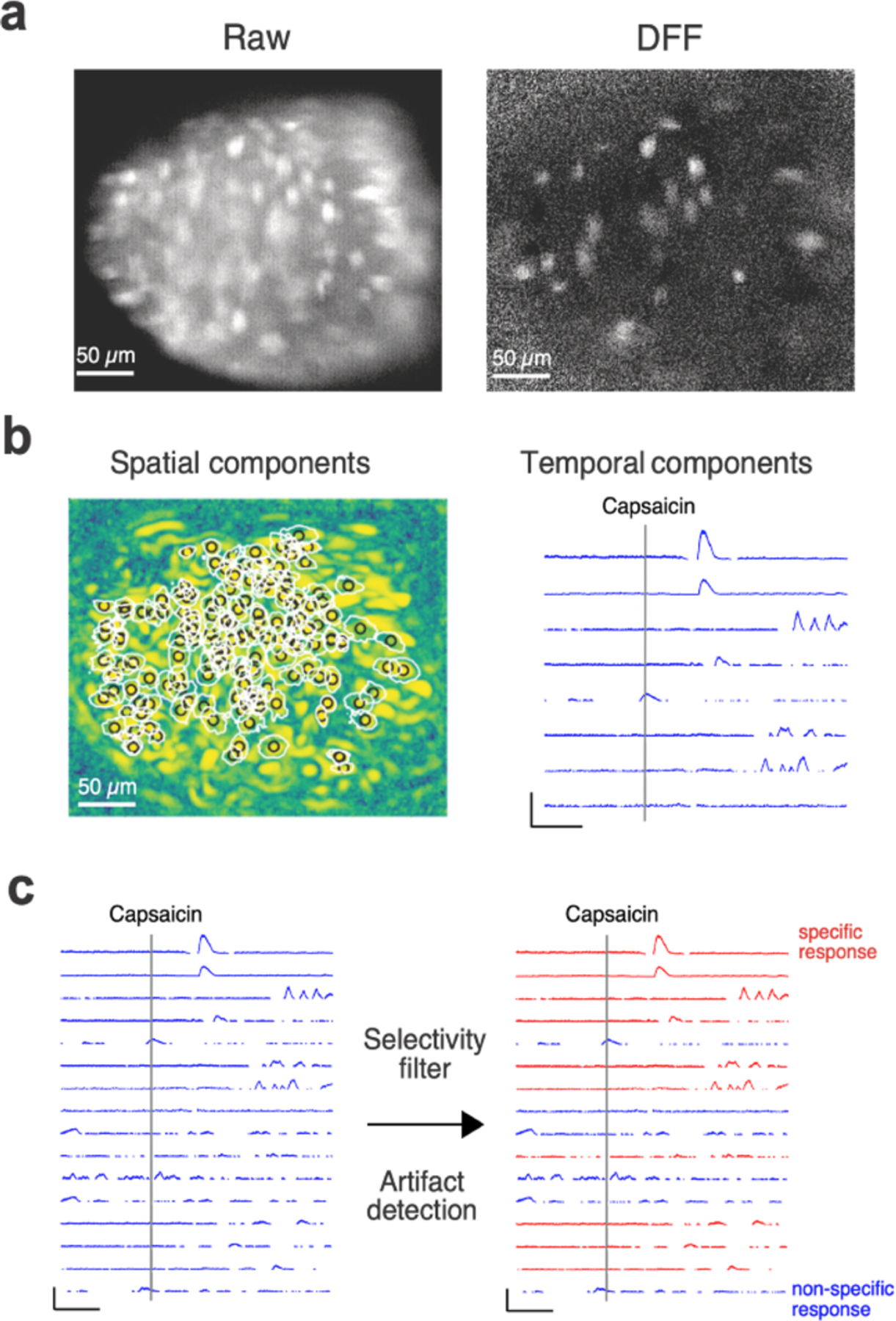
Identification of stimulus-specific responses. (a) Representative image frames from calcium imaging experiments of the jugular-nodose ganglion. Left, raw data depicting high background fluorescence at 20 fps. Right, frame captured at the same time point with a DFF filter demonstrating activity in individual neurons. (b) Left, region of interest map showing the detection of individual neurons as spatial components. Right, representative traces of calcium transients (temporal components) in sensory neurons of the jugular-nodose ganglion. Scale bar, DFF 100, 25 s. (c) Left, calcium traces showing multiple neuronal responders to the stimulus (capsaicin) delivered at the timepoint represented by the vertical line. Right, the same calcium transients are filtered by the specificity of the response to the stimulus and following artifact detection. Stimulus-specific responses are labeled in red, while non-specific responses are labeled in blue. Scale bars, DFF 100, 25 s.

Viewing the software from the live feed, the Miniscope was lowered via a stereotaxic arm to obtain a stable optical plane of visible neurons (figure 2(a)). To guide the lens when lowering the Miniscope, the LED light was targeted onto the center of the jugular-nodose ganglia. Continuous monitoring of the Miniscope live feed at this step revealed the intact ganglion structure, with individual neurons and vasculature visible. Upon making superficial contact with the ganglia surface, the bottom lens surface was lowered until slightly depressing the ganglia to maximize the number of cells in the optical plane. Light contact with the ganglia is also crucial for stabilizing the ganglia complex from breathing-associated movement artifacts. Continued adjustments in the horizontal and vertical planes were made to center and focus the ganglionic surface in the optical plane. To maximize the number of cells in the field of view, the efferent axon tracks were typically positioned away from the recording surface. Changing to the delta fluorescence over baseline fluorescence (DFF) filter feed on the acquisition software should display single-neuron resolution activity (figure 2(a)). Occasional repositioning of the ganglia on the mesh retractor may be necessary to reduce obstructing vasculature or to optimize the number of active neurons within the field of view.

Common issues encountered in the physical Miniscope setup included motion artifacts, shifting of the liquid medium continuum, and vasculature bleeding. Blood flow through the vasculature may be erroneously registered as neural activity, therefore, logging potential sources of error within the recording field of view is helpful during analysis. Motion artifacts could appear as abrupt jolting of the ganglia, usually corresponding to the breathing pattern of the mouse. These disruptions result in intense DFF noise that obscures individual neuron activity for several seconds. Motion artifacts are typically attributed to tension on the vagus nerve, transmitting vibrations from chest movements onto the ganglionic complex, or the mesh retractor making contact with the salivary glands. Sufficient space (~2 mm) between the retractor and chest surface should be maintained to prevent breathing fluctuations from inducing motion artifacts. To alleviate tension on the vagus nerve, the retractor can be lowered or re-positioned to reduce tension, given that space between the retractor and tissue is maintained. If possible, connective tissue covering the pectoral muscles can be gently removed to lengthen the freely-moving portion of the vagus nerve.

Upon completion of the ganglia isolation and placement onto retractor, anesthesia levels for recordings were generally maintained at 1.0%–1.25% isoflurane in 100% oxygen with a flow rate of 1.5 l min ^1^. The respiratory rates are stabilized between 100 and 150 breaths per minute. Artifacts induced by increased respiratory rate changes are addressed by increasing isoflurane levels, whereas gasping-induced artifacts are adjusted via lowering the anesthetic concentration.

Shifting of the liquid continuum can produce a border edge artifact that can move into the optical frame and potentially obscure the ganglia. Repositioning the retractor at an angle so that it is not horizontal may aid in avoiding this type of edge effect. In these situations, the Miniscope will have to be stereotactically raised and re-focused, and the angle of the retractor will have to be adjusted to be at approximately 0°. Once the Miniscope has been re-focused, the retractor should be flushed with saline to re-establish the liquid continuum. Some mice will have vasculature near the ganglia that result in superficial bleeding on the ganglionic surface and can occasionally obscure neural activity. Forceps can be used to gently massage out blood present in the vessels of the ganglia, followed by a saline wash. If the bleeding continues to obstruct the view, the ganglion can be rotated to position the vasculature onto the bottom and out of the field of view. Note, that all adjustments must be followed up by assessing the Miniscope live view focus for single neuron resolution. Upon achieving stabilization of the raw feed and confirming single neuron activity, calcium activity recording can be started.

For chemical agonist experiments in this study, the recording paradigm was initiated with a 30-min baseline period to minimize activity associated with mechanical stimulation from the isolation and focusing process. Typically, over the baseline period, mechanical activity was high and decreased over time. Solutions were directly applied to the exposed vagus nerve as 15 L droplets using a 20 L micropipette (3123000039 Eppendorf Research Plus, Enfield, CT) (figure 1(f)). Saline applications preceded agonist applications as a negative control for mechanical stimulation. Saline application was used as a wash between different stimuli.

### 3.2. Motion correction and DFF steps

All actions were performed in an ipynb notebook (Jupyter Notebook). We first applied NoRMCorre to motion correct the half-sized TIFFs. Motion correction is necessary to keep a steady image for proper ROI detection and temporal trace output that is not contaminated by movement. Within CaImAn, a package called NormCorre is used for motion correction. NormCorre is performed as previously described by (Pnevmatikakis and Giovannucci 2017). A Fast-Fourier Transform is performed frame-by-frame looking for strong phase correlation to determine the amount of *x*–*y* translation. Depending on the motion, rigid motion only or rigid with piecewise patch, spatial window motion correction is used. The images are exported and assessed by a user to determine optimal stabilization.

The neurons in the periphery imaged by the 1-photon (1P) Miniscope device have varying levels of calcium fluorescence across entire recordings. Therefore, we normalized the calcium data as the change in fluorescence. This is calculated by a normalized change in fluorescence, ([dF/F] or DFF for short) filter. To achieve this we used the movies.movie.computeDFF() function, native to CaImAn, to calculate the DFF of the movie. We applied a filter that calculates the DFF of each pixel in the image after motion correction is performed. This is done by binning the data into 5-second segments to calculate baseline activity within the pixel. Changes above the 20th percentile of the pixel fluorescence intensity distribution within this time window are displayed/detected as positive DFF activity. The images were manually inspected after the filter to note and remove any potential artifacts, either by removing bad frames or false positive ROIs. Application of the DFF improved contrast sufficiently to visually confirm neural activity (figure 2(a)).

### 3.3. ROI detection

Once motion correction and the DFF filter were applied, we analyzed the movie dataset of interest using specific source extraction and deconvolution parameters in the constrained nonnegative matrix factorization for microEndoscopes (CNMF-E) pipeline Zhou *et al* 2018. The CNMF-E pipeline is designed for 1P data, such as that collected by the Miniscope. The CNMF-E pipeline does not intrinsically incorporate a DFF calculation. Therefore implementation of the DFF prior to CNMF-E improved processing of ganglion data, which has high background signal and high contrast. The result of this computation is two matrices. One with the spatial components of the detected neuron ROIs, and a second with the temporal components of the mean fluorescence within each ROI over time (figure 2(b)). We saved these matrices into a pickle file for subsequent analysis. We also developed an optional pipeline that can fix (maintain) the generated ROIs for different video inputs. This could be potentially useful for tracking a known population of neurons in a larger video dataset.

### 3.4. Analysis steps

Once calcium traces from the neurons detected in the recording were computed and saved, we needed a method to extract the important information. We developed a custom analysis pipeline to quantify how many calcium transients there were in each trace and calculate features of these transients such as peak amplitude, duration, number of peaks, rise time (slope), decay time, and area under the curve (response integral), rise (slope), and decay (slope). Calcium transients were detected by finding peaks in the traces that had peak amplitudes three standard deviations above the mean baseline fluorescence (figure 2(c)). This pipeline also has a selectivity filter to detect which neurons responded to a stimulus that was applied during the recording. The function determines which neurons responded specifically to the stimulus within a specific time frame and outputs all of the aforementioned data points about each calcium transient within the stimulus window for the responding neurons. Responders were determined by identifying neurons with detected calcium transients within the pre-determined post-stimulus window. A neuron was excluded from being a responder if the maximum amplitude of activity during the pre-stimulus control window was greater than 50% of the post-stimulus maximum amplitude. The traces and properties of the responding neurons were saved into comma-separated values files for statistical analysis.

Another feature of the custom analysis pipeline is an artifact detection function. During experimental recordings, we occasionally observed calcium transients that were present in every neuron at the same time in the recording. Because the experiments are conducted *in vivo*, there are instances of movement primarily related to mouse breathing. While this is minimized by the retractor platform being decoupled from the body, there are still occasional instances of movement. The artifact detection function automatically detects these instances in the data and excludes them from being mistaken for real neural activity. This is accomplished by flagging frames in which 30% of the neurons have a peak within a 1 s time window (figure 2(c)). There is also a function to plot the neurons detected as responders and non-responders in these plots to ensure that the function is working as intended.

### 3.5. CaImAn processing

**Figure 3.**
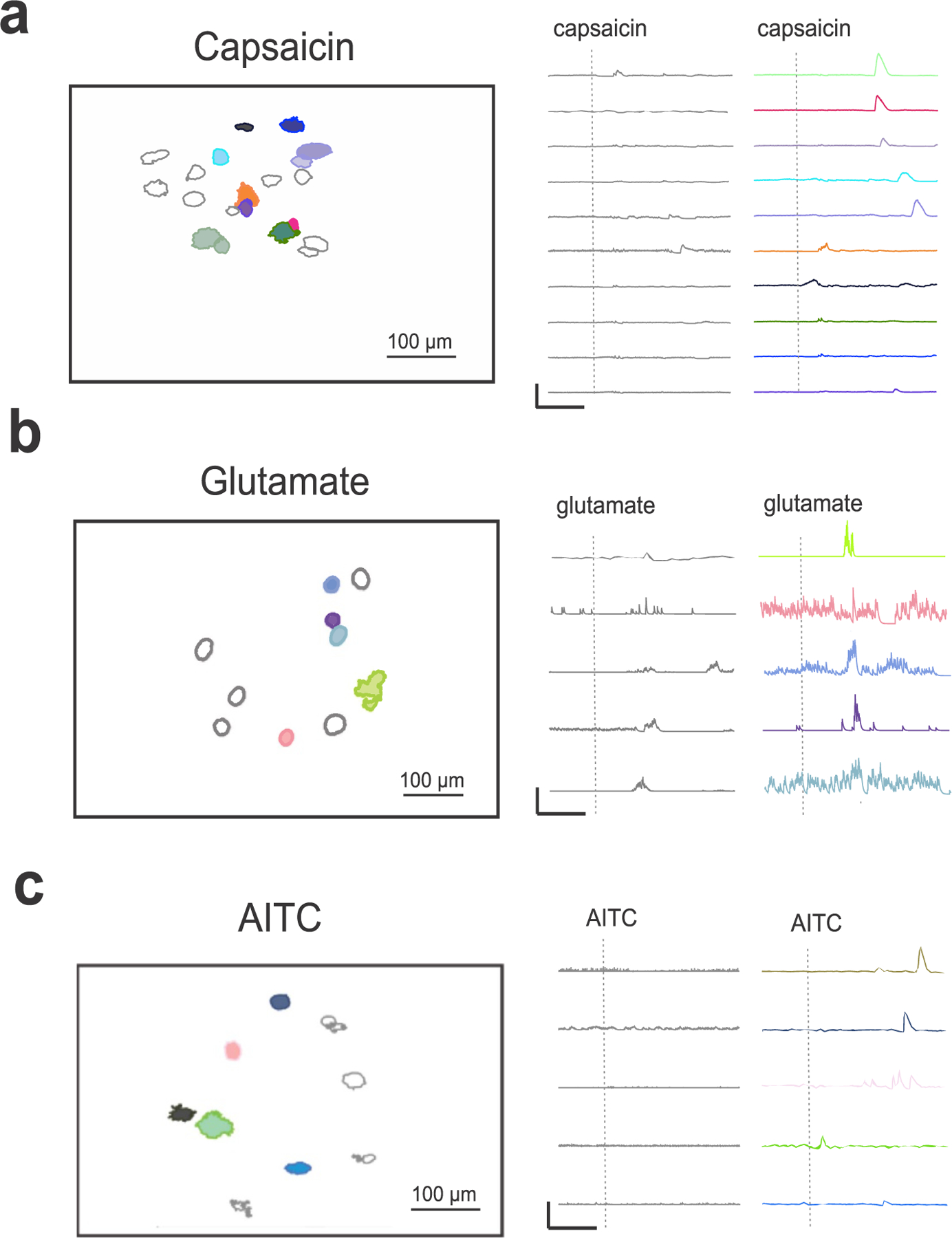
Representative stimulus-specific responses. (a) Left, spatial ROIs of responsive jugular-nodose ganglion sensory neurons within 100 sec of an application of capsaicin on the vagus nerve. Right, color-coded corresponding calcium traces matching the spatial ROIs in response to capsaicin application (denoted by the vertical line). Grey spatial ROIs and traces represent non-specific responders and responses that were not sufficient to pass the selectivity filter. Scale bar, 250 DFF, 50 s. (b) Left, spatial ROIs of jugular-nodose ganglion sensory neurons in response to glutamate application on the vagus nerve. Right, corresponding calcium traces to the glutamate responders. Scale bar, 50 DFF, 50 s. (c) Left, spatial ROIs to AITC application on the vagus nerve. Right, corresponding calcium traces in response to AITC application. Scale bar, 250 DFF, 50 s.

Generally, parameters provided by CaImAn that affect region-of-interest (ROI) detection based on neuron/pixel size are in-line with suggestions provided by the CaImAn tips and tricks section (https://caiman.readthedocs.io/en/master/CaImAn_Tips.html). gSig and gSiz are kept at 6 and 25 pixels respectively, with a half-size patch and stride of 36 and 25 pixels respectively. Downsampling is kept at a minimum, with a maximum of a factor of 2 spatial or temporal downsampling. Parameters to threshold ROIs based on minimum pixel spatial correlation and peak-to-noise ratio vary depending on the images but typically are in the range of 0.6–0.9 and 4–10 respectively. Merge thresholding is typically very high (0.999 ) to prevent proximal neurons from blending after the DFF calculation. Usually, a full background is sufficient for proper ROI detection. While we do use the convolution neural network to assist in accepting/rejecting detected ROIs as neural activity, we manually inspect each of those ROIs to determine if it is a false positive or false negative. This is supported by the selectivity filter in the post analysis (figure 2(c)). Detected neurons can be plotted spatially as ROIs along with their corresponding calcium traces (figures 2(b) and 3).

### 3.6. Jugular-nodose ganglia sensory neurons respond distinctly to stimuli applied to the vagus nerve

With our recording platform and analysis pipeline, responses to specific stimuli can be plotted as individual ROIs (neurons) and their corresponding calcium traces (DFF). Within the time frame of 3000 frames (150 s), multiple responses were recorded to the transient receptor potential vanilloid 1 (TRPV1) agonist capsaicin when applied to the vagus nerve (figure 3(a)). Capsaicin application to the vagus nerve evoked several specific neuronal responders (color-matched traces to spatial ROIs), while other neurons had non-specific responses (labeled in grey color). Application of the neurotransmitter glutamate on the vagus nerve also evoked neuronal responses in the 150 s time window (figure 3(b)). Vagus nerve application of the transient receptor potential ankyrin 1 (TRPA1) channel agonist, allyl isothiocyanate (AITC) produced a subset of specific responses (figure 3(c)). Comparison between the calcium traces in response to different stimuli revealed the amplitude scale (DFF) for capsaicin responses and AITC responses were larger than glutamate (figure 3).

**Figure 4.**
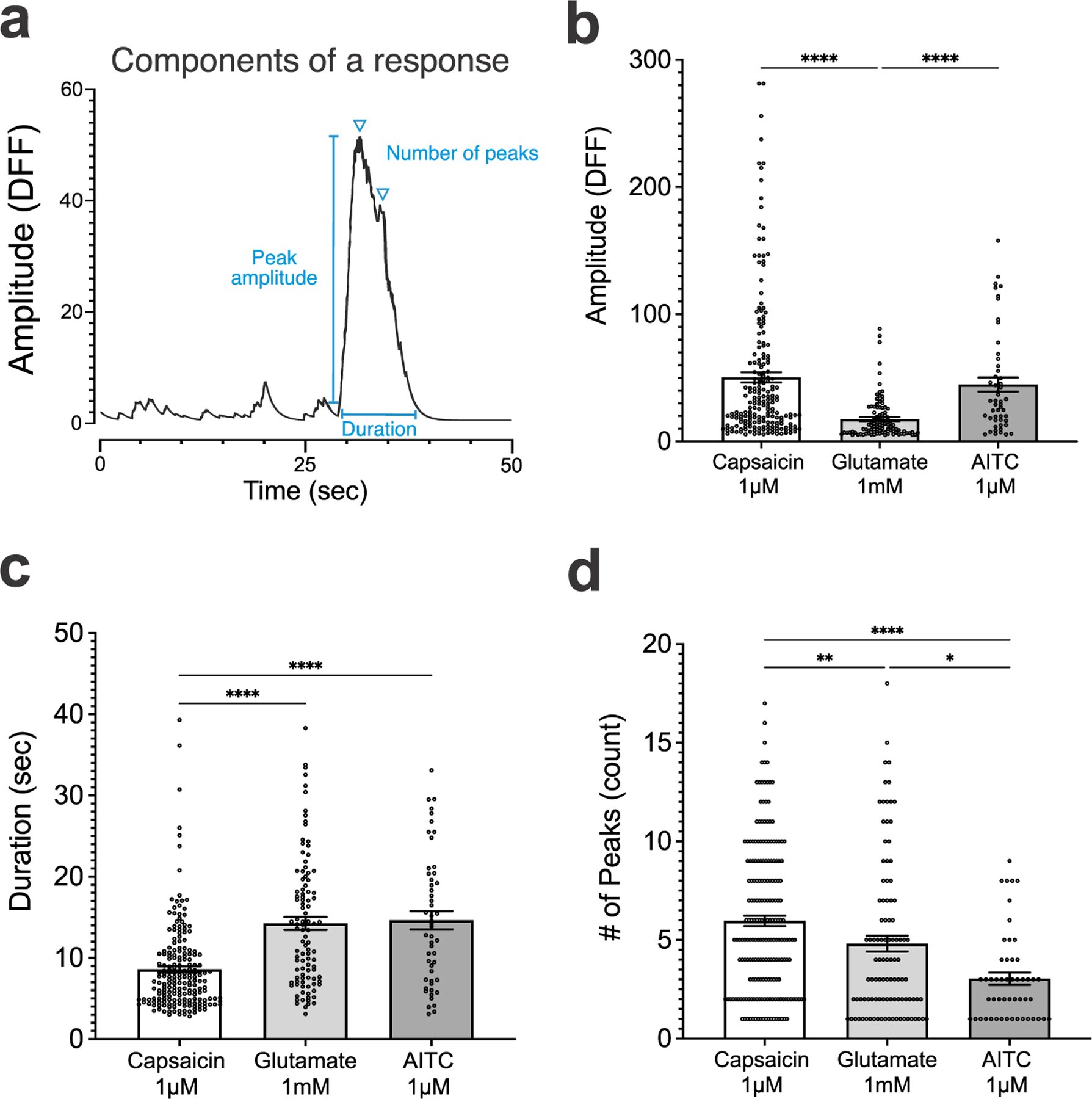
Quantification of stimulus-specific calcium transients. (a) Features of individual calcium transients that are extracted for further analysis: peak amplitude (DFF), duration (s), number of peaks (count). (b) Comparison between capsaicin, AITC, and glutamate application reveals that capsaicin- and AITC-specific responses have a significantly larger peak amplitude than glutamate. Each data point in the scatterplot represents the calcium transient response from a single ROI. Bar plots show group mean values and standard error of the mean. (c) Comparison between the duration of capsaicin, AITC, and glutamate responses demonstrates that glutamate and AITC responses are significantly longer than capsaicin responses. (d) Quantification of the number of peaks in response to the application of capsaicin, AITC, and glutamate reveals that the three stimuli induce significantly different number of peaks for each respective stimuli. Capsaicin responses were found to have the largest number of peaks, while AITC responses had the lowest.

To quantify the calcium transients, we extracted multiple features of the response, such as amplitude (DFF), duration (s), and the number of peaks per response (count; figure 4(a)). The stimuli-specific responses were quantified in terms of these components, aggregated across experiments, and statistically compared between stimuli. A comparison of the peak amplitude across the three stimuli revealed that capsaicin and AITC responses were significantly larger than glutamate responses (figure 4(b), DFF, capsaicin vs glutamate: 50.40 ± 4.007 vs 17.74 ± 1.627, *P* < 0.0001, Kruskal–Wallis test; AITC vs glutamate: 44.75 ± 5.518 vs 17.74 ± 1.627, *P* < 0.0001, Kruskal–Wallis test). There was no significant difference found between the amplitude of capsaicin and AITC responses (figure 4(b), capsaicin vs AITC: 50.40 ± 4.007 vs 44.75 ± 5.518, *P* > 0.999, Kruskal–Wallis test). This indicates that the peak amount of fluorescence, which is correlated with calcium influx and neural activity, was significantly elevated in capsaicin and AITC responses compared to glutamate. We also compared the duration of the responses and found that capsaicin responses were significantly shorter than AITC or glutamate (figure 4(c), capsaicin vs glutamate, 8.588 ± 0.3836 vs 14.25 ± 0.7881, *P* < 0.0001; capsaicin vs AITC: 8.588 ± 0.3836 vs 14.63 ± 1.130, *P* < 0.0001, Kruskal–Wallis test). The response duration between AITC and glutamate was not significantly different (figure 4 c, 14.25 ± 0.7881 vs 14.63 ± 1.130, *P* > 0.9999, Kruskal–Wallis). These data demonstrate that specific stimuli applied to the vagus nerve induce periods of neural activity for varying amounts of time depending on the stimulus. Another feature that can be quantified from calcium transients is the number of peaks for a response. Capsaicin has a significantly larger number of peaks compared to glutamate (figure 4(d), count, 5.965 ± 0.2634 vs 4.814 ± 0.3979, *P* = 0.0043, Kruskal–Wallis test), and a significantly larger number of peaks when compared to AITC responses (figure 4(d), count, 5.965 ± 0.2634 vs 3.039 ± 0.3131, *P* < 0.0001, Kruskal–Wallis). Glutamate responses were found to have significantly more number peaks than AITC responders as well (figure 4(d), count, 4.814 ± 0.3979 vs 3.039 ± 0.3131, *P* = 0.0304, Kruskal–Wallis test). The significant differences between the number of peaks for specific stimuli suggests that individual neurons have a signature neural activity pattern triggered by a particular stimuli on the vagus nerve.

**Figure 5.**
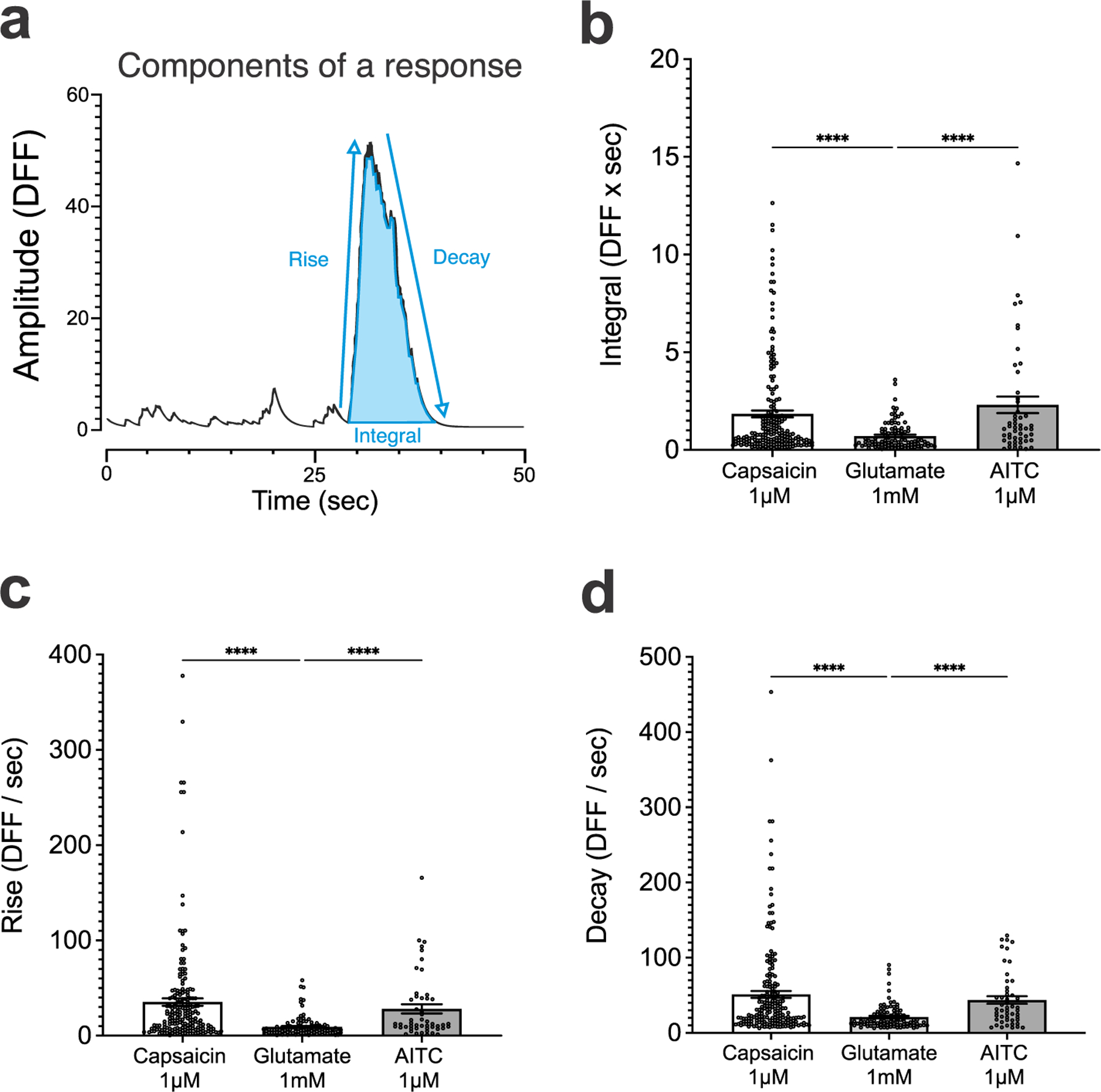
Second order quantification of stimulus-specific calcium transients. (a) Second order features of individual calcium transients that are extracted for further analysis: integral (DFF s), rise (DFF/s), delay (DFF/s). (b) Comparison between the area under the curve of responses triggered by application of capsaicin, glutamate, and AITC reveals that capsaicin and AITC specific responses have a significantly larger integral of response than glutamate specific responses. Each data point in the scatterplot represents the calcium transient response from a single ROI. Bar plots show the group mean values and standard error of the mean. (c) Comparison between the rise (upward slope of response) demonstrates that capsaicin and AITC have significantly larger rise than glutamate responses. (d) Quantification of the decay (downward slope of response) reveals that capsaicin and AITC have larger decay than glutamate specific responses.

We also quantified second order components of the calcium transients including integral (DFF s), rise (upward slope of response, DFF/s), and decay (downward slope of response, DFF/s) (figure 5(a)). The integral of the response was defined as the area under the curve of the stimulus-specific response. Comparison of the integral between capsaicin and glutamate responses demonstrated that capsaicin integrals were significantly larger than glutamate integrals (figure 5(b), DFF s, 1.842 ± 0.1755 vs 0.7094 ± 0.0692, *P* < 0.0001, Kruskal–Wallis test), while AITC response integrals were larger than glutamate (figure 5(b), DFF x sec, 2.306 ± 0.4232 vs 0.7094 ± 0.0692, *P* < 0.0001, Kruskal–Wallis test). No significant difference was found between capsaicin and AITC integrals (figure 5(b), DFF s, 1.842 ± 0.1755 vs 2.306 ± 0.4232, *P* = 0.7261, Kruskal–Wallis test). We analyzed the rise of each response across our stimulus by measuring the slope of the response between the start of the response until the time of peak amplitude. We found that both capsaicin and AITC rise responses were significantly larger than glutamate (figure 5(c), DFF/sec, capsaicin vs glutamate: 35.28 ± 3.881 vs 9.263 ± 1.056, *P* < 0.0001; AITC vs glutamate: 28.08 ± 4.832 vs 9.263 ± 1.056, *P* < 0.0001, Kruskal–Wallis test). No significant difference was found between the rise of capsaicin and AITC responses (figure 5(c), DFF/s, 35.28 ± 3.881 vs 28.08 ± 4.832, *P* > 0.999, Kruskal–Wallis test). Finally, we determined the decay of each response as the absolute value of the slope of the response starting at the time of peak amplitude until the end of the response. Both capsaicin and AITC responses had significantly larger decay than glutamate responses (figure 5(d), DFF/sec, capsaicin vs glutamate, 51.05 ± 4.520 vs 20.92 ± 1.586, *P* < 0.0001; AITC vs glutamate, 43.62 ± 5.079 vs 20.92 ± 1.586, *P* < 0.0001, Kruskal–Wallis test). No significant difference was found between capsaicin response decay and the AITC group (figure 5(d), DFF/s, 51.05 ± 4.520 vs 43.62 ± 5.079, *P* > 0.999, Kruskal–Wallis test). Taken together, these second order component comparison distinguish marked differences in the dynamics of stimulus-specific responses to canonical activators of TRPV1 and TRPA1 channels (capsaicin and AITC, respectively), compared to activation by glutamate.

### 3.7. Calcium transients evoked by nerve-applied agonists can be used for random forest model classification

Sensory stimuli transduced by receptors on the nerve, or at nerve terminals, are encoded by neural activity at the cell body. To determine whether the recorded vagal sensory neuron calcium transients could be used to identify the type of stimulus applied to the nerve, we used a random forest classifier, which is a supervised machine learning algorithm that is commonly used for classification purposes (Breiman 2001). Our lab has previously used machine learning approaches to identify cytokine-specific responses from vagus nerve-recorded compound action potentials (Zanos *et al* 2018). Here, our goal was to determine whether the neural activity from individual vagal sensory neurons by itself could be used to identify the three different agonists applied to the vagus nerve.

**Figure 6.**
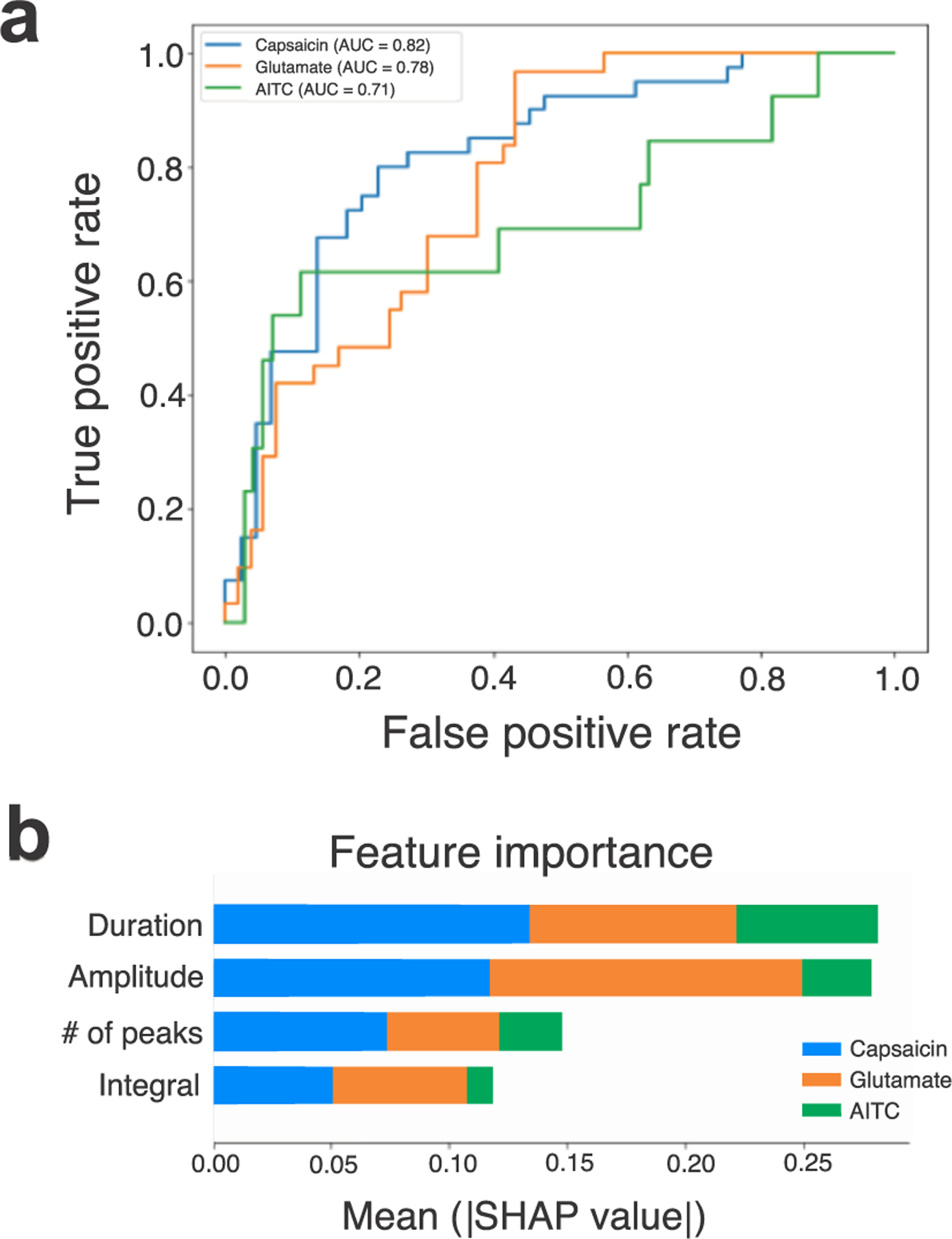
Random forest classifier identifies subsets of vagal sensory neuron responders based on calcium transient activity. (a) Receiver operating characteristics (ROC) curve showing classification performance for each class of agonist. (b) Feature importance based on SHAP values indicates that the amplitude and duration had the largest impact on the model output.

We used a random forest model (scikit-learn; Pedregosa *et al* 2011) with ten-fold cross-validation on multiple calculated features of the calcium transients (peak amplitude, duration, numbers of peaks, and integral). Using the three types of chemical stimuli as the classes, the model input was responses to capsaicin (*n* = 200), glutamate (n = 102), and AITC (*n* = 51). The random forest model had a mean classification accuracy of 71 ± 4.9% (mean ± SD) with relative performance of the three classes shown on the receiver operating characteristic (ROC) curves (figure 6(a)). To examine the importance of each transient feature on the output of the model, we used SHapley Additive exPlanations (SHAP; Lundberg and Lee 2017). The average impact on of all the inputs on the model output, as measured by SHAP values, indicated that the amplitude and duration of the transients were the most important predictors across the models (figure 6(b)). This result suggests that the encoding of these specific stimuli may be based on the shape of the transients, rather than second-order measures, at least in this dataset.

### 3.8. Jugular-nodose ganglia immunohistochemistry

**Figure 7.**
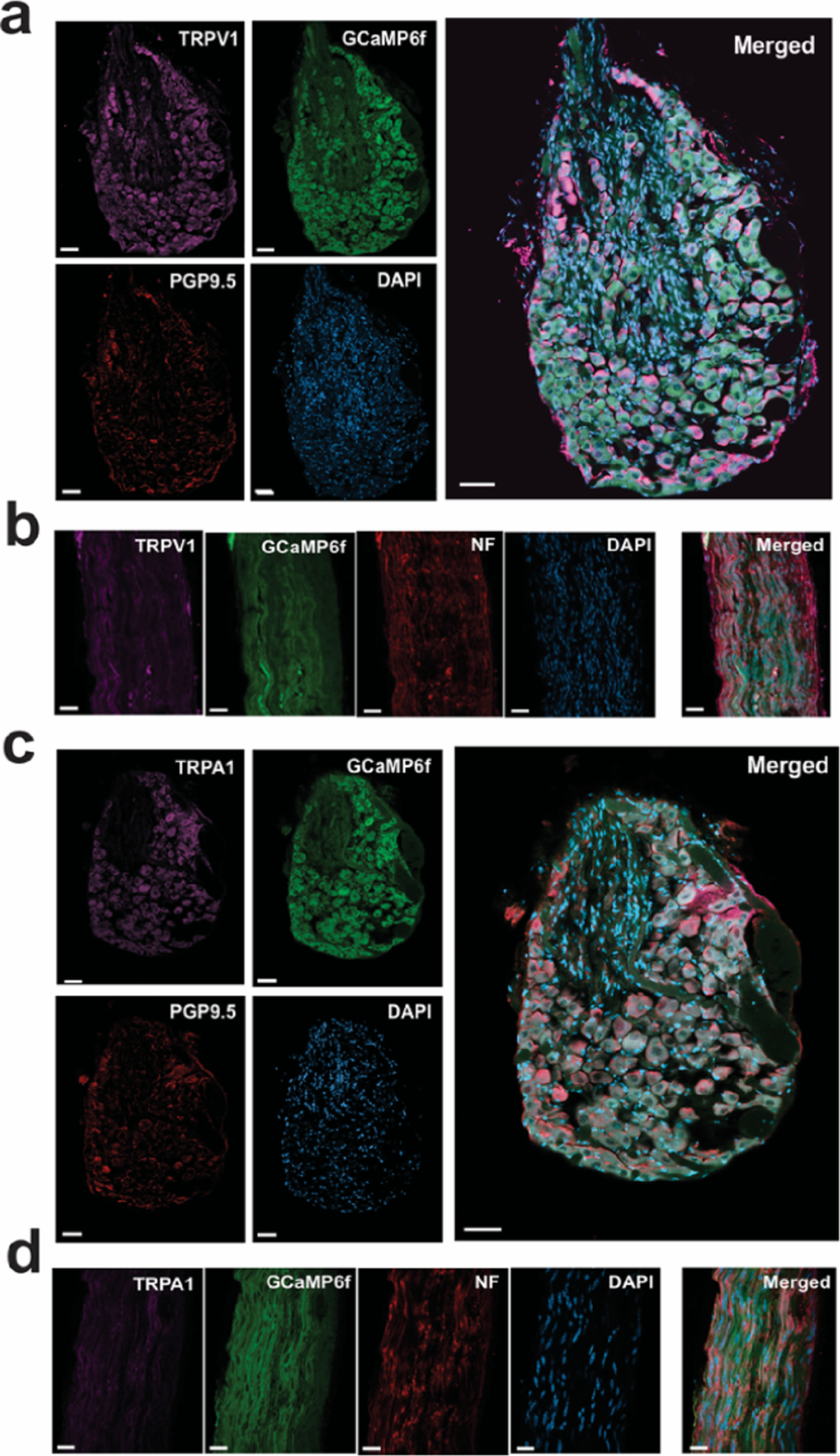
Immunohistochemistry of jugular-nodose ganglia and vagus nerve from Vglut2-GCaMP6f mice showing presence of TRPV1 and TRPA1. (a) Representative confocal microscopy images of jugular-nodose ganglia with fluorescent labeling of TRPV1, PGP9.5, and DAPI. Scale bar, 50 m. (b) Representative images of the cervical vagus nerve with fluorescent labeling of TRPV1, neurofilament, and DAPI. Scale bar, 20 m. (c) Representative images of the jugular-nodose ganglia showing TRPA1 labeling. (d) Representative images of the cervical vagus nerve showing TRPA1 labeling. Vglut2-GCaMP6f signal is endogenous in all samples. Note, regions of the ganglia where neurons are absent contain the efferent vagus nerve fibers that pass through the ganglia.

To verify the presence of GCaMP6f and TRP channels in the ganglia, we performed immunohistochemistry on samples harvested following calcium imaging. To label peripheral neurons, we used antibodies to PGP9.5, which is highly expressed in peripheral neurons and their projections (LeMaster *et al* 1999). The vast majority of jugular-nodose ganglia neurons are glutamatergic, as identified by the overlap of PGP9.5 labeling and the endogenous Vglut2-GCaMP6f signal (figures 7(a) and (c)). This is consistent with other studies of these ganglia (Chang *et al* 2015, Kupari *et al* 2019). To confirm the presence of TRPV1 and TRPA1 channels, we used validated polyclonal antibodies to label these ion channels on both the jugular-nodose ganglia and the vagus nerve, where we directly applied the chemical agonists during our experiments. TRPA1 and TRPV1 were visualized on both the cell bodies in the ganglia (figures 7(a) and (c)) and on the vagus nerve (figures 7(b) and (d)). The differential distribution of TRPA1 and TRPV1 is also in line with previously reported patterns for these TRP channels in rodents (Jawaid *et al* 2022). To visualize the vagus nerve fibers, we co-labeled with a monoclonal antibody to neurofilament, which is found abundantly in axons (Yuan *et al* 2012). Note that the region of the ganglia where neurons are absent is where the vagal parasympathetic efferent fibers cross through the interior of the ganglia descending to the viscera (figures 7(a) and (c)).

## 4. Discussion

In this study, we have outlined a surgical method and analysis pipeline to acquire and analyze *in vivo* calcium imaging data from the mouse jugular-nodose ganglia. We used this technique to show that chemical stimuli applied to the vagus nerve elicit sensory neuron activation that can be measured using calcium imaging, quantified, and then used to accurately classify distinct subsets of neuronal responders.

Previous work from our lab demonstrated that the amplitude and shape of compound action potentials recorded from the vagus nerve were important parameters that suggested sensory neurons encode information about visceral stimuli by these primary features, rather than by firing rate or integral (Zanos *et al* 2018). Here, in this study, we show that specific features of neuronal activity recorded from individual vagal sensory neurons are important for classifying different subsets of cells. The high feature importance of the response amplitude and duration, as measured by SHAP values (figure 6(b)), suggests that vagal sensory neurons use initial firing rates and sustained firing to encode the type of stimuli present at the nerve or terminal level. Further work will be needed to determine what other encoding strategies, such as graded or combinatorial coding, may be used by these neurons to represent other types of stimuli (Wang *et al* 2018). While fluorescent calcium indicators such as GCaMP6 do not report single action potentials with complete fidelity, the high sensitivity and reliability of these indicators indicates that they are an excellent proxy for neural activity (Dana *et al* 2014, Huang *et al* 2021). Therefore, we believe that optical electrophysiology will accelerate our ability to connect large-scale neural activity to ongoing physiology in the PNS. Machine learning approaches leveraging classification tools and neural networks will also help to achieve a better understanding of the information content within recorded PNS signals (Zanos 2019, Glaser *et al* 2020, Koh *et al* 2020).

We have focused on imaging the jugular-nodose ganglia with a Miniscope system that is designed to be mounted onto a hard stable surface, such as the skull, for awake-behaving imaging in rodents. Due to the cervical location of the ganglia *in situ*, there is not a rigid surface for mounting the Miniscope base-plate. Located at the base of the skull, where the vagus nerve exits the cranium to innervate the viscera, the jugular-nodose ganglia are challenging to access (Han and de Araujo 2021), which is why we have outlined this surgical approach. While we have focused on this specific PNS ganglion in the cervical region of the mouse, we believe that the surgical approach and analysis pipeline demonstrated here can be generalized and applied to other sites in the rodent PNS, such as the trigeminal ganglia, DRG, celiac ganglia, and others. The outlined approach provides an experimental platform for using optical electrophysiology, in this case with genetically encoded calcium indicators, to monitor neural activity in small peripheral ganglia that are often located in soft tissue within the body.

Some limitations of our approach include the fact that these experiments were performed under anesthesia, and that severing the nodose connections on the cranial side may affect the health status of the jugular-nodose ganglia. As general anesthetics have known effects on ligand-gated ion channels and other confounds (Dilger 2002), avoiding anesthesia would be desirable, however, this remains a significant obstacle given the location of the jugular-nodose ganglia at the base of the skull and in a highly dynamic region of the neck. Saline application was used to keep the nerve moist throughout the recording, but there may be possible improvements for further enhancing the stability of the jugular-nodose ganglion neurons. For example, we have utilized a mesh retractor as a platform in this study, but other approaches such as using agar or an engineered silicone support could be viable alternatives. If more chronic approaches are developed for imaging, a biocompatible semi-rigid compartment to stabilize the ganglion would be needed to control for movement artifacts. Continued development and stabilization strategies may enable chronic awake-behaving imaging in the future, which would provide an improved measure of structure-function relationships in neuromodulation and bioelectronic-based approaches (Chang 2021). In regards to our machine learning classifier, there are also important constraints to be considered. Our random forest classification results indicate that the specific agonist applied to the vagus nerve can be accurately identified based on features of the calcium transients collected in this sample set (figure 6), but this may not generalize well to other data sets. Additional neural activity data in the context of other types of stimuli, chemical or otherwise, are needed to build a more robust model for the type of encoding used by these from these vagal sensory neurons.

The utilization of minature calcium imaging microscopes and neural activity reporters in whole peripheral ganglia allows access to a range of new applications within the PNS and visceral targets. For example, some types of immune cells (e.g. pancreatic beta cells, Merkel cells) are especially difficult to access with traditional electrophysiological tools, such as patch-clamp or rigid electrodes. As the sophistication of imaging sensors and activity reporters improves over time, researchers will be able to answer new mechanistic questions that connect macroscopic network-level activity to more microscopic compartments of the nervous system, such as *in vivo* axonal activity (Broussard and Petreanu 2021) and neurotransmitter release (Marvin *et al* 2019). Further, as neuromodulation approaches focused on the PNS continue to expand (Johnson and Wilson 2018, Kaye *et al* 2021), the development of tools and techniques to precisely monitor neural activity in the periphery will be critical (Guiraud *et al* 2016, Garrett *et al* 2021). Glutamatergic neuron activity, such as that measured here in Vglut2-GCaMP6f mice, in the CNS and PNS is a primary form of excitatory communication and the ability to assess transmission and plasticity in neural circuits is of critical importance for the control of many physiological functions (Browning and Travagli 2010, Chang *et al* 2019). Newly developed monitoring tools that include optical electrophysiology and high-density recording probes, such as Neuropixels (Steinmetz *et al* 2021), will likely play an important role in the validation of a new generation of noninvasive nerve modulation technologies. For instance, high-intensity focused ultrasound (Cotero *et al* 2020, Huerta *et al* 2021, Hoffman *et al* 2022), electromagnetic modulation (Cywiak *et al* 2020), and optogenetic approaches (Michoud *et al* 2021) are being increasingly utilized on PNS targets.

In this study, we utilized the Miniscope hardware platform with customized open-source software packages (i.e. CaImAn) and Python-based post-hoc analyses to suit our specific laboratory aims of studying vagal sensory neuron responses to different chemical stimuli applied to the vagus nerve. By directly imaging the cell bodies of the vagal sensory afferents that carry important sensory information from the viscera to the brain, we have shown that different types of vagus nerve-applied stimuli evoke distinct responses from sensory neurons that can be discriminated with sufficient accuracy using a random forest classifier. Our results provide insight into the encoding strategies that vagal sensory neurons utilize to transmit information about noxious stimuli or chemical agonists to the brain. This novel framework enables an improved understanding of vagus nerve signaling that may also be relevant to many neuromodulation-based approaches targeting this nerve (Pelot and Grill 2020, Mughrabi *et al* 2021 Ahmed *et al* 2022). These types of tools and software analyses strengthen the ongoing efforts of multiple lab groups to decode and understand the physiological signals carried by complex mixed nerves, such as the vagus nerve (Guiraud *et al* 2016, Setell *et al* 2020, Vallone *et al* 2021). We find it likely that similar approaches, using a new generation of optical tools and genetic reporters, will enable broad access to a variety of new neural targets within the PNS.

## Figures and Tables

**Table 1.** List of resources and equipment used in this study.

Reagent or resource	Source	Catalog number
Tools, chemicals, and antibodies		
Isoflurane, USP	Covetrus	11 695-6777-2
Miniscope (UCLA)	Labmaker	V3.2
Small Animal Physiological Monitoring System	Harvard Apparatus	75–1500
Nair Hair Remover Aloe Vera/ Lanolin Lotion	Church & Dwight	2709 004
Fisherbrand Sterile Alcohol Prep Pad	Fisher Scientific	22-363-750
Micro Dissecting Scissors	Roboz Surgical Instruments Co	RS-5912SC
Hemostatic Forceps	Roboz Surgical Instruments Co	RS-7111
No.7 Forceps	Roboz Surgical Instruments Co	RS-5947
No.7 Vessel Dilation Forceps	Roboz Surgical Instruments Co	RS-4927
Retractor Elastomer	Fine Science Tools	18200-07
Blunt Retractor	Fine Science Tools	18200-09
Micro Dissecting Spring Scissors	Roboz Surgical Instruments Co	RS-5602
EZFlow cell strainer	Foxx Life Sciences	410-0001-OEM
Paraformaldehyde solution 4% in PBS	Santa Cruz Biotechnology	CAS 30525-89-4
Sucrose	Sigma Aldrich	S0389
Cryostat	Leica	CM1850
10% Normal Goat Serum	Abcam	ab190565
Triton X-100	Thermo Scientific	A16046.AP
Alexa Fluor 647 recombinant rabbit monoclonal anti-Neuronal Nuclei	Abcam	EPR12763
DAPI Fluoromount Mounting Medium	Southern Biotech	0100-20
Flurorescent Confocal Microscope	Zeiss	LSM 880
Experimental models: mouse strains		
Mouse: VGlut2-ires-cre (C57BL/6 J)	The Jackson Laboratory	28863
Mouse: ROSA-GCaMP6f aka Ai95D (C57BL/6 J)	The Jackson Laboratory	28865
Mouse: VGlut2-GCaMP6f	Bred In-house	

## Data Availability

All data that support the findings of this study are included within the article (and any supplementary files).
